# The case for a HRQL core outcome set: outcome reporting bias in oesophageal cancer studies

**DOI:** 10.1186/1745-6215-12-S1-A77

**Published:** 2011-12-13

**Authors:** Rhiannon C Macefield, Marc Jacobs, Natalie S Blencowe, Ida J Korfage, Joanna Nicklin, Sara T Brookes, Mirjam A Sprangers, Jane M Blazeby

**Affiliations:** 1 School of Social and Community Medicine, University of Bristol, Bristol, BS8 2PS, UK; 2 Department of Medical Psychology, Academic Medical Center/University of Amsterdam, 1105 AZ Amsterdam, The Netherlands; 3 Department of Public Health, Erasmus MC, 3000 CA Rotterdam, The Netherlands

## Objectives

The large number of scales and items within generic and disease specific HRQL measures may lead to outcome reporting bias of HRQL data. We explored this hypothesis in publications of HRQL results from studies of curative treatment for oesophageal cancer.

## Methods

Systematic literature searches (MEDLINE, Embase, PsycINFO and CINAHL) identified articles reporting HRQL data after curative treatments for oesophageal cancer from validated patient-completed questionnaires. The most frequently used instruments were examined (EORTC QLQ-C30, QLQ-OES24, QLQ-OES18, SF-36 & FACT-E). Outcome reporting bias was explored by examining which of the instrument’s scales and single items were documented in the methods and the results of papers, with each article checked by two independent reviewers.

## Results

Of 52 included papers, three specified to focus on a restricted set of the measured scales in the study methods, with one providing a rationale for doing so. 31 papers (60%) reported EORTC QLQ-C30 results, of which 20 (65%) reported all 15 of the instrument’s scales and single items. Global health status was consistently reported (30/31 papers, 97%), followed by physical function (29/31, 94%) and role function (28/31, 90%). Insomnia and constipation were least reported (22/31, 71%). 25 papers additionally included the QLQ-OES24 or QLQ-OES18 oesophageal-specific module (consisting of 11 and 10 scales and single items respectively). All of the module scales and items were reported in 14/25 papers (56%). Dysphagia, eating and reflux scales were most often reported (in 24, 23, and 22 papers respectively). 12 papers reported SF-36 results, of which 9 (75%) reported all 8 scales. SF-36 physical functioning, role-physical, role-emotional, social functioning, vitality and general health scales were reported most often (11/12 papers), followed by bodily pain and mental health (10/12 papers). Three studies used the FACT-E, and all reported the 5 subscales (physical well-being, functional well-being, social/family well-being, emotional well-being and the oesophageal cancer subscale).

## Conclusions

Selective outcome reporting was evident in some studies of curative treatment for oesophageal cancer, increasingly when a higher number of scales and items were available. The development of a core outcome set of HRQL domains for RCTs of curative treatments in oesophageal cancer may reduce the risk of outcome reporting bias and ensure important domains are consistently reported.

